# The Outcomes of Adult Acquired Buried Penis Surgical Reconstruction

**DOI:** 10.3390/life14101321

**Published:** 2024-10-17

**Authors:** Marco Falcone, Natalia Plamadeala, Lorenzo Cirigliano, Mirko Preto, Federica Peretti, Ilaria Ferro, Martina Scavone, Emanuele Zupo, Paolo Gontero

**Affiliations:** 1 Urology Clinic—A.O.U. “Città della Salute e della Scienza”—Molinette Hospital, University of Turin, 10126 Turin, Italy; marco.falcone@unito.it (M.F.); lorenzo.cirigliano@unito.it (L.C.); mirko.preto@unito.it (M.P.); federica.peretti@unito.it (F.P.); ilaria.ferro@unito.it (I.F.); martina.scavone@unito.it (M.S.); emanuele.zupo@unito.it (E.Z.); paolo.gontero@unito.it (P.G.); 2 Neurourology Clinic—A.O.U. “Città della Salute e della Scienza”—Unità Spinale Unipolare, 10126 Turin, Italy; 3 Urology Department, Medical Faculty, Biruni University, Istanbul 34015, Turkey

**Keywords:** adult acquired buried penis, penile reconstruction, skin grafts, patient-reported outcome, surgical and functional outcomes

## Abstract

Adult Acquired Buried Penis (AABP) is a morbid condition that often requires surgical intervention. This retrospective study of 46 patients who underwent AABP surgery from November 2017 to July 2023 evaluates surgical outcomes, functional outcomes, and patient-reported outcomes. The median follow-up (FU) was 46 months. Patients were categorized by surgical complexity using the Pariser classification, with 76.1% undergoing high-complexity procedures (Pariser ≥ III). Common comorbidities included obesity (58.7%), prior circumcision (52.2%), and hypertension (52.2%). The low-complexity group had a shorter hospital stay (*p* = 0.02). No other significant differences were noted between groups in terms of Body Mass Index, operative time, or FU. Sexual dysfunction (45.7%) and urinary issues (38.1%) were the main reasons for surgical consultation. Skin grafting was required in 63.0% of patients; partial graft loss was more common in full thicknes skin graft group (*p* = 0.04). Postoperative complications occurred in 32.6% of patients, 13.3% of which were classified severe (Clavien ≥ III). The median increase in stretched penile length was 2 cm. The recurrence rate was 21.7%. The 12-month recurrence-free survival rate was 89.1%. All groups saw significant improvements in urinary and sexual function post-surgery (*p* < 0.05), and high patient satisfaction was reported (90.3%). Despite the complication rate, AABP surgery significantly improves quality of life, with ongoing advancements in technique anticipated to enhance outcomes further.

## 1. Introduction

Adult Acquired Buried Penis (AABP) was initially described by Dr. Keyes in 1917. This morbid condition is characterized by the entrapment of the penis within the surrounding peri-genital tissues secondary to a chronic inflammatory state [1]. Various factors, including genital Lichen Sclerosus (LS), prior radical circumcision, and concurrent comorbidities such as diabetes mellitus (DM, 7–56%), obstructive sleep apnea syndrome (OSAS), hypertension (7–59%), and smoking (0–54.2%), may contribute to the onset or exacerbation of AABP [2,3,4,5,6,7]. However, the main risk factor for developing AABP is obesity. As obesity rates are rising in Western countries, with 27.9% of adults having an abnormal Body Mass Index (BMI), buried penis incidence is following similar epidemiological patterns. Consequently, reconstructive urologists must cope with a growing demand for surgical intervention [7,8,9].

Irrespective of the cause, AABP can lead to debilitating consequences, including urinary dribbling, inflammation, skin breakdowns, and infections. Patients frequently encounter difficulties in regard to maintaining genital hygiene, esthetic discomfort, erectile dysfunction (ED) (with painful erections), and a higher risk of developing penile cancer. Additionally, it can cause psychological distress, low self-esteem, and depression [10,11,12,13].

Surgical intervention becomes imperative when conservative treatments fail to manage the disorder [14]. Surgical options encompass a broad spectrum of procedures. As there is no consensus regarding the preferred classification system, the choice of classification remains at the discretion of individual medical centers [15,16,17,18,19,20]. Pariser et al. [18] categorized the surgical procedures for addressing AABP into five groups based on the degree of technical complexity, ranging from simple to complex interventions. These include circumcision with penile degloving, penile shaft reconstruction using skin flaps, full-thickness grafts (FTSGs), split-thickness grafts (STSGs), or scrotal flaps for significant penile skin loss; scrotoplasty; excision of the suprapubic fat pad (escutcheonectomy); and abdominoplasty. Some additional techniques include the incision of the fundiform and/or suspensory ligament of the penis, as well as the fixation of the dorsal skin of the penis to the pubic periosteum. The objectives of these surgical repairs are to expose the penile shaft, reconstruct the penile teguments, and remove abnormal genital or abdominal tissues (including skin, subcutaneous tissues, or fat) contributing to the dartos layer’s pathological degeneration. The overarching goals are to reduce the risk of AABP recurrence, improve patients’ quality of life, and minimize surgical complications.

This study aims to describe surgical and functional outcomes, determine recurrence-free survival (RFS) rates, and investigate various outcomes and risk factors through subgroup analysis following the surgical management of AABP in order to provide a comprehensive understanding of the effectiveness of different surgical approaches, identify patient characteristics associated with better or worse outcomes, and, ultimately, improve clinical decision making and patient care in the management of AABP.

## 2. Materials and Methods

This single-center observational study retrospectively reviewed patients diagnosed with AABP who underwent their first surgical management for an acquired buried penis between November 2017 and July 2023. The primary outcome of interest was determining the recurrence rate at FU and the RFS at the 12-month follow-up. Recurrence was defined as the presence of clinical recurrence of AABP, regardless of whether patients chose to pursue further treatment or not. The secondary outcomes aimed to describe and analyze patient demographics, etiologies, surgical procedures, intra- and postoperative complications (using the Clavien–Dindo classification) [21], and functional outcomes related to erectile and urinary function following penile reconstruction.

Surgical treatment for AABP was considered necessary when conservative approaches were unsuccessful and led to issues such as impaired personal hygiene, urinary or sexual difficulties, psychological distress, or reduced self-esteem and quality of life. The choice of surgical method was tailored to each patient and considered factors like comorbidities, anesthesiologic risk, the presence of abdominal or suprapubic fat pads, and the quality and availability of penile skin. Preoperative assessments were conducted to evaluate the quality of abdominal, thigh, and penile skin. The surgeon determined tissue viability both before and during surgery. If the penile tissue was non-viable or there was insufficient skin to cover the shaft after debridement, reconstruction using skin grafts was mandatory. At our institution, FTSGs were typically utilized when performing procedures such as escutcheonectomies or abdominoplasties, whilst STSGs were preferred whenever less invasive procedures were performed or abdominal skin was unsuitable for grafting. Measurements of the flaccid stretched penis size were taken both pre- and postoperatively, measuring from the pubic bone to the tip of the glans with the foreskin retracted to ensure the shaft was fully stretched.

**Figure 1:**
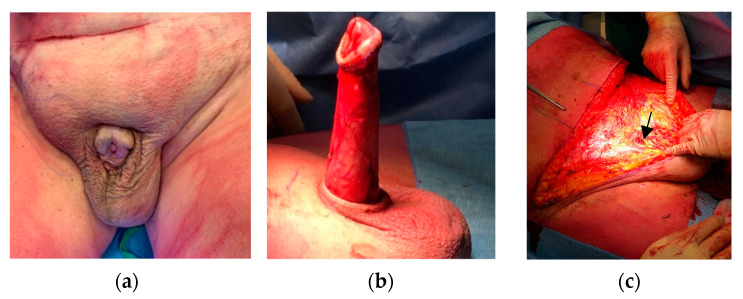

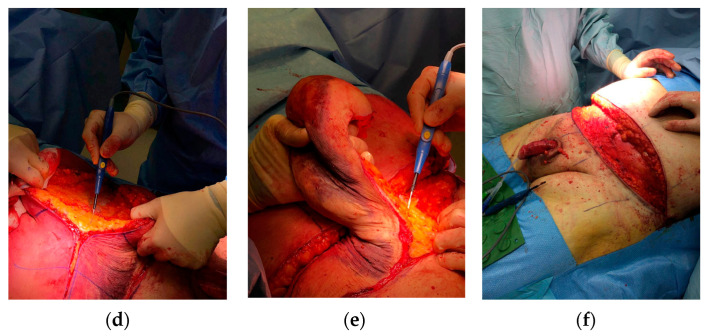
Step-by-step surgical approach employed in this study: (**a**) Patient position before surgery. The patient lies in the supine position. (**b**) Degloving the penis after following circumferential incision around the glans. Note that dissection goes down to the penopubic junction until complete liberation is achieved. (**c**) Incision of fundiform (black arrow) and suspensory ligaments and fixation of skin to the pubic periosteum. (**d**–**f**) Removal of prepubic and abdominal fat.

**Figure 2:**
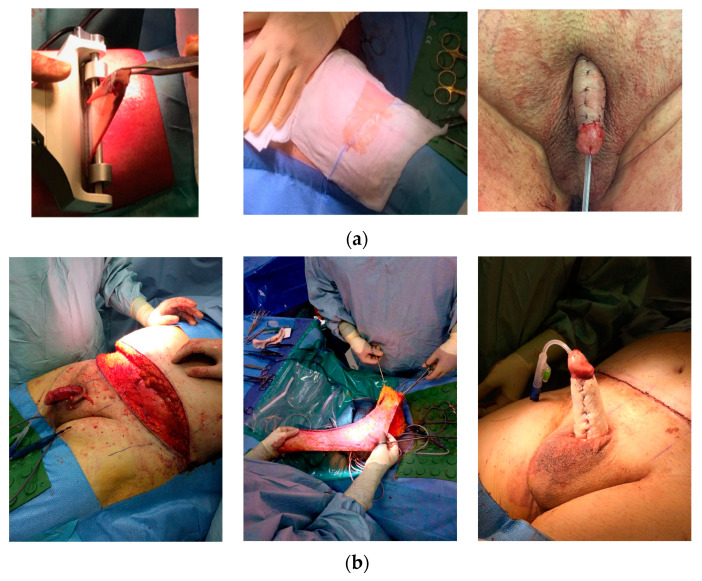
Skin graft harvesting and use: (**a**) The split-thickness skin graft (STSG) is obtained from the anterior thigh using an air dermatome with 0.3 mm thickness. The donor site is prepared with Vaseline). (**b**) For a full-thickness skin graft (FTSG), removed escutcheon or abdominal tissue is used.

Surgical procedures were classified into five categories based on technical complexity following the Pariser–Santucci classification [18]: penile shaft debridement and reconstruction with local flaps (category I), penile shaft reconstruction with skin grafts (category II), scrotal debridement or scrotoplasty (category III), escutcheonectomy/fat pad excision (category IV), or complete abdominal panniculectomy/apronectomy (category V). Patients who underwent multiple surgical procedures were classified according to the higher grade performed. The most utilized techniques are summarized as follows (Figure 1):

- Patient Positioning: Supine position and adequate draping, with the abdomen/thighs exposed for skin graft harvesting (Figure 1a).
- Penile Shaft Debridement/Unburying: A circumferential incision is fashioned around the glans and a subsequent degloving of the penis is carried out, carefully dissecting down to the penopubic junction until complete liberation is achieved. The use of a glans stitch can provide traction during the procedure (Figure 1b).
- Assessment of Penile Skin Quality: If necessary, penile skin and dartos fascia are removed leaving the Buck fascia as a vascularized bed for an FTSG, STSG, or scrotal flaps.
- Penopubic Ligament Fixation: To enhance penile exteriorization, in some cases, fundiform and suspensory ligament incisions are performed, and the skin is fixed to the pubic periosteum using PDS*II 2/0 sutures (Ethicon Inc., a subsidiary of Johnson & Johnson, Somerville, NJ, USA) (Figure 1c).
- Removal of Prepubic/Abdominal Fat: Prepubic fat can be removed through either a pubic lipectomy or pubic liposuction. For morbidly obese patients with significant abdominal adiposity, an abdominal panniculectomy may be performed concurrently. In our center, the assistance of a plastic surgeon is not paramount. (Figure 1d–f)
- Drain Placement and Foley Catheter: Appropriate use of drains to prevent a postoperative hematoma and seroma formation is crucial. A suction drain is usually left in place for the first 48 h after surgery. Additionally, a Foley catheter is placed during the patient’s hospitalization.
- Harvesting the Skin Graft or Using Local Flaps: The skin graft for covering the penile shaft with an STSG is obtained from the anterior thigh using an air dermatome. The donor site is prepared with Vaseline. We prefer a graft thickness of 0.3 mm, as it more closely mimics the characteristics of penile skin (Figure 2a). For an FTSG, escutcheon or abdominal tissue is used, with the harvested graft being defatted to remove excess subcutaneous fat (Figure 2b). The graft is then secured to the underlying Buck’s fascia of the recipient site with Monosyn 4/0 quilting sutures (B. Braun, Melsungen AG, Melsungen, Germany) to optimize graft take. Quilting sutures are applied longitudinally, away from neurovascular bundle vascular pedicles, to minimize the risk of glans ischemia.

At the end of the procedure, a compressive penile dressing and an indwelling urethral catheter were maintained for one week to ensure optimal graft take. We typically employ a bolster dressing to protect the wound, support proper healing, and provide stability during the recovery period. The bolster dressing comprises several layers: The innermost layer is petrolatum gauze to prevent the dressing from sticking to the wound. This is followed by a layer of gauze or dacron wool soaked in a saline and mineral oil mixture placed on top of the petrolatum gauze and secured with Vicryl 0 (Ethicon Inc., a subsidiary of Johnson & Johnson, Somerville, NJ, USA) stay sutures to keep it in place. The third layer consists of sterile gauze for cushioning and wound protection. The outermost layer is Peha-haft (Hartmann, Heidenheim, Germany) dressing, which offers compression and support. Patients were discharged from the hospital once the dressing and catheter were removed. Starting two weeks postoperatively, patients were instructed to apply topical hyaluronic acid and vitamin complexes to minimize scar contracture during the healing process. Follow-up appointments were scheduled at one week and then at 1, 3, and 6 months after surgery to monitor patient recovery and progress.

To assess sexual function before and after surgery, the validated International Index of Erectile Function (IIEF-15) [22] was used at baseline and again 12 months postoperatively. Lower-urinary tract symptoms were evaluated using the validated International Prostate Symptom Score (IPSS) both before and after surgery [23,24]. The IPSS includes seven questions addressing voiding and storage symptoms. Scores range from 0 to 7 for mild symptoms, 8 to 19 for moderate symptoms, and 20 to 35 for severe symptoms. Additionally, patient-reported outcomes were assessed 12 months postoperatively using a specially created 6-item questionnaire (see Table A1).

The normality of variable distributions was tested by the Kolmogorov–Smirnov test. Categorical variables were described using frequencies and percentages. Differences between groups were assessed by a chi-square test or Fisher’s exact test depending on the expected cell counts. For continuous variables with non-normal distribution, the median and interquartile range (IQR) were reported. Differences between groups were assessed by a Mann–Whitney U test for non-normally distributed variables. Logistic regression and linear regression were used to identify risk factors for complications and recurrence. The RFS rate over time was estimated using a Kaplan–Meier analysis. A log-rank test was applied to identify any significant differences in survival between groups. The Wilcoxon test was used to calculate *p*-values for comparing pre- and post-surgery IIEF-5 scores and IPSSs between the groups. A *p*-value < 0.05 was considered statistically significant. Statistical analyses were performed using the Statistical Package for the Social Sciences (SPSS; v. 29; IBM, Chicago, IL, USA)

## 3. Results

### 3.1. Study and Patients’ Characteristics

A total of 46 patients with AABP were eligible for the current study. The patient demographics are summarized in Table 1. The median age was 60.5 years (IQR 50.0–71.0). The most common risk factor was obesity, with 95.5% of patients having a BMI > 25 kg/m^2^, followed by a history of previous radical circumcision (52.2%). The median BMI was 30.0 kg/m^2^ (IQR 28.0–35.0). Of these patients, 29 (63.0%) required penile skin grafts (STSGs in 69.0% and FTSGs in the remaining 31.0%). High-complexity reconstructive surgery (Pariser category ≥ III) was performed in most cases (76.1%). A statistically significant difference was observed regarding overweight and obese (BMI > 25 kg/m^2^) patients, who were more numerous in the high-surgical-complexity group (*p* = 0.01). However, in terms of obesity alone (BMI ≥ 30 kg/m^2^), there were no differences between the two groups. No significant difference was seen between the groups regarding their operative time and follow-up, except for the hospital stay, which was lower in the low-complexity reconstruction group (*p* = 0.02).

The primary indications for surgery were as follows: obesity with the burying of the shaft in the peri-genital adipose tissue in 25 patients (54.3%); lichen sclerosus (LS) in 10 patients (21.7%); severe phimosis after previous radical circumcision in five patients (10.9%); previous penile cancer surgery in four patients (8.7%); and genital lymphedema/granuloma in two patients (4.4%). Table 2 demonstrates the inter-group differences in etiologies and major complaints regarding the complexity of the surgery. The major issues requiring surgical consultation reported by the patients were sexual problems (*n* = 21, 45.7%), urinary problems (*n* = 18, 38.1%), poor personal hygiene (*n* = 4, 8.7%), and esthetic issues (*n* = 3, 6.5%), with no statistically significant differences being seen between groups.

The surgical features related to skin graft use are summarized in Table 3. An STSG was the preferred option for shaft reconstruction, used in 69.9% of patients requiring skin grafting. All STSGs were harvested from the anteromedial thigh using an air dermatome, while most FTSGs (88.9%) were harvested from abdominal skin. Complete graft loss occurred in one case (3.4%) in the STSG group. Partial graft loss was significantly higher in the FTSG group (*p* = 0.04). No other statistically significant differences were identified between the groups in terms of skin graft complications.

### 3.2. Surgical Outcomes

**Figure 3:**
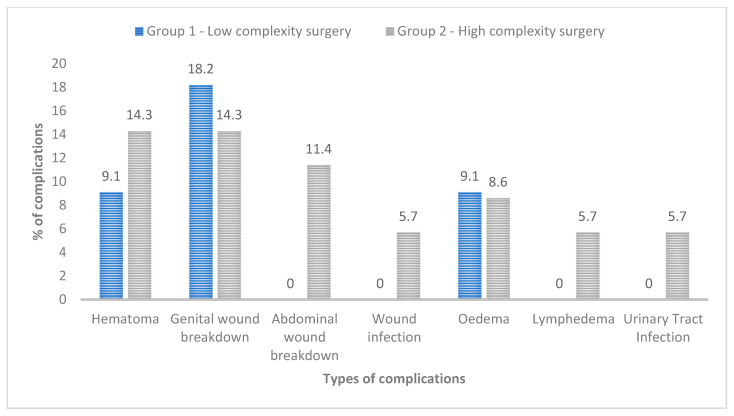
Overall postoperative complications according to the type of surgery.

Table 4 shows the surgical outcome measures. Overall, 32.6% of patients experienced postoperative complications. Most of the complications were minor (Clavien–Dindo grade < III), while 13.3% had major complications requiring surgical revision (Clavien–Dindo grade ≥ III), all of which occurred in the high-complexity group. The major complications (grade IIIb) included genital wound breakdown with complete loss of the STSG, which necessitated surgical debridement, and hematoma formation in the FTSG group, which required surgical evacuation. No other high-grade complications (grades IV and V) were reported. Figure 3 represents all post-surgical complications observed during the follow-up, noting that some patients experienced more than one complication simultaneously. The recurrence rate at follow-up was 21.7%. The overall RFS rate at 12 months was 89.1%. No statistically significant difference was found between the two groups in terms of recurrence rate. In our cohort, all patients showed a significant improvement in penile stretched length after surgery (*p* < 0.01). The median preoperative length was 7 cm (IQR 6.0–9.6) compared to a postoperative median length of 11.3 cm (IQR 8.5–13.0), reflecting a median increase of 2 cm. However, no significant difference in the increase in penile length was observed between the groups. Both the low-complexity and high-complexity surgical groups showed significant improvement in stretched penis size at the six-month follow-up. The low-complexity group improved from a median of 7 cm (IQR 4–10.5 cm) to 12 cm (IQR 9–13 cm), while the high-complexity group improved from 7 cm (IQR 6.0–9.3 cm) to 10 cm (IQR 8.3–12.5 cm), with *p* < 0.01 for both groups.

### 3.3. Functional Outcomes

For the functional outcomes, 31 patients (67.4%) completed the questionnaires (Table 5). The IPSS showed a significant improvement in urinary function in both surgical groups (*p* < 0.05). The IIEF-15 score also indicated a significant enhancement in sexual function post-surgery (*p* < 0.05). No significant differences were found between the low- and high-complexity surgery groups concerning preoperative and postoperative IPSS or IIEF-15 scores. However, the STSG group had significantly lower preoperative and postoperative IIEF-15 scores than the FTSG group (*p* = 0.002). Most patients (over 66.7% in each domain) reported improvements in patient-reported outcomes (PROs) across all domains, with an overall satisfaction rate of 90.3% and an improvement in quality of life of 93.5% (Table 6).

## 4. Discussion

Buried penis was initially classified as a congenital condition predominantly affecting children. However, with the increasing prevalence of metabolic syndrome and obesity worldwide, it has emerged as a significant pathological condition in adults, substantially impacting their quality of life [7,8,9].

The surgical management of AABP presents a complex challenge due to the multifactorial etiology of the condition, patient comorbidities, and the varying degrees of penile burial. The results of this study provide valuable insights into the outcomes of different surgical approaches, particularly in the context of recurrence rates, complications, and functional improvements in erectile and urinary function [20].

As an increasingly emerging condition, the data currently available in the literature are limited by relatively short follow-up periods, with an average of 17.7 months. Our study, however, is distinguished by a longer follow-up period, with a median of 46 months.

Given the broad clinical presentation and the range of surgical options available for treating AABP, several classification systems have been proposed in the literature that are either based on preoperative assessment [16,17] or surgical techniques [18,19]. However, standardization is lacking. In our study, we applied the Pariser–Santucci classification [18], a simplified system introduced in 2018 that comprises five categories, is organized according to surgical complexity, and differentiates between low-complexity (<III) and high-complexity (≥III) repair procedures. The high percentage (76.1%) of patients undergoing high-complexity procedures reflects the mostly severe nature of cases managed at our center. Additionally, we observed a significantly longer hospital stay in the high-complexity group compared to the lower-complexity group (4 days versus 3 days, *p* = 0.02). This extended stay can be attributed to higher Clavien III postoperative complication rates, which necessitate additional monitoring and interventions, as well as the presence of more severe pre-existing conditions or comorbidities. Pariser et al. observed similar percentages (69%) of patients undergoing complex repairs in their study [18]. The choice of complex surgical techniques underscores the necessity of a tailored approach based on individual patient anatomy and comorbid conditions. Therefore, the development of high-level evidence-based studies is crucial for enhancing our understanding of AABP, improving patient counseling, and facilitating comprehensive management strategies, particularly given the complexities associated with reconstructive genital surgery.

The high prevalence of overweight and obesity in our cohort (95.5% with a BMI over 25 kg/m^2^ and 58.7% with a BMI ≥ 30 kg/m^2^) underscores obesity as a major risk factor for AABP, supporting previous research that identifies obesity as a key contributor to this condition due to increasing abdominal girth [5,15,16,18,25,26,27]. In some studies, BMI values even reach up to 55 kg/m^2^, further emphasizing the strong link between AABP and obesity [13]. The presence of hypertension (52.2%), diabetes mellitus (26.1%), and active smoking (39.1%) further highlights the potential association between AABP and metabolic syndrome. Additionally, a history of previous surgeries, such as radical circumcision (52.2%) or penile cancer surgery (15.2%), may reinforce the existing literature suggesting that these procedures contribute to the development of AABP through scar tissue formation or lymphedema, leading to the progressive entrapment of the shaft into the peri-genital tissues [4,28,29].

The recurrence rate of 21.7% at follow-up highlights the need for ongoing monitoring and potentially adjunctive treatments to manage recurrent cases. The overall recurrence-free survival (RFS) rates of 89.1% at 12 months are promising, suggesting that most patients achieve sustained benefits from surgery, though recurrence remains a concern for a significant minority. When considering the one-year RFS rate based on surgical complexity, similar values were found when comparing the less complex surgical group to those with high complexity (90.9% vs. 88.6%, respectively). Previous studies have reported comparable success rates ranging from 85% to 100%. Pariser et al. found that, after AABP surgery, the low-complexity group had a higher RFS rate than the high-complexity group (100% vs. 86%, no significant difference), with an overall success rate of 91% [18]. Similarly, Hampson et al. [30] reported an 85% success rate, while Tausch et al. [19] reported an 88% success rate. These findings suggest that a well-tailored procedure for the patient may result in a high surgical success rate. In our study, BMI was not found to be a predictive factor for recurrence.

The observed postoperative complication rate of 32.6% is consistent with the complexity of the surgeries performed and is comparable to complication rates reported in other studies, where it reached up to 80.8% [16,18,27,31,32]. While most complications were manageable conservatively (Clavien < III), two patients (13.3%) required surgical revision (Clavien ≥ III), all occurring in the high-complexity group. In particular, one patient experienced progressive anemia after surgery with the formation of an inguinoscrotal hematoma, necessitating surgical drainage (Clavien III b), while another patient required surgical curettage due to complete graft loss and wound dehiscence with infection in the genital area (Clavien III b). This indicates that, while these procedures can effectively address AABP, they are not without risk. Although our multivariate analysis did not reveal any correlation between BMI ≥ 30, smoking, hypertension, or type of surgery and complications, the literature highlights high BMI, smoking, surgical complexity, and additional procedures like abdominoplasty as primary risk factors for complications following buried penis repair surgery [18,30,32]. It is essential to recognize that patients with elevated BMI often present with comorbidities that can adversely affect wound healing and increase the risk of postoperative infections. This may explain the higher complication rates observed in American studies where the median BMI frequently exceeds 40 kg/m^2^. Variations in patient characteristics can necessitate different surgical approaches; for instance, patients with severe obesity may require more aggressive interventions, which carry an increased risk of complications. In particular, Aubé et al. [32], in a study on 24 patients with AABP who underwent surgical treatment, identified BMI ≥ 40 and tobacco smoking as independent predictors of overall complications, with hazard ratios of 25 and 14.6, respectively. They reported an overall complication rate of 62.5%, with 29% classified as high-grade complications (Clavien ≥ III). Hampson et al. [30] reported a postoperative complication rate of 33%, with high-grade complications occurring in 17% of cases. In their multivariate analysis, they found that BMI was the only factor significantly associated with complications, with each unit increase in BMI raising the odds of complications by 10%. Similarly, Pariser et al. [18] noted an overall complication rate of 65%, with high-grade complications (Clavien ≥ III) occurring in 23% of patients, all of whom were in the high-complexity group (*p* = 0.02). In contrast, our cohort includes a wider variety of patient profiles, enabling us to examine a range of surgical procedures along with their corresponding surgical and functional outcomes. This diversity may help explain the variations in complication rates and outcomes when compared to studies that primarily involve severely obese patients.

When evaluating candidates for AABP surgery, it is vital to balance the expected benefits with potential risks. Important considerations include a comprehensive medical history, effective patient counseling, and treatment at a high-volume center. Due to the significant risk of high-grade postoperative complications, a multidisciplinary approach is recommended to optimize outcomes and manage any issues that may arise. We strongly recommend weight loss through pharmacological means or bariatric surgery, along with smoking cessation at least six weeks before surgery in order to minimize the risk of complications and enhance overall surgical outcomes.

Skin grafting is a crucial component of AABP surgical management, particularly for penile shaft reconstruction. In this study, STSGs were used in 69.9% of cases, showing a higher graft take rate compared to FTSGs (95% versus 90%, respectively), which is consistent with the existing literature [4,15,33]. The preference for STSGs can also be attributed to the frequent occurrence of lichen sclerosus (LS), identified in 54.5% of cases in our previous study [23], necessitating skin grafting and reflecting the complexity of our patient cohort and the extensive need for penile resection. LS often renders surrounding tissues unsuitable for FTSG harvesting. Monn et al. [34], on the other hand, reported that FTSGs demonstrated successful graft takes. Similarly, the study by Gul et al. [35] found no significant differences in outcomes between STSGs and FTSGs. These findings suggest that both FTSGs and STSGs are valid and effective options for penile shaft reconstruction regardless of the underlying etiology.

In the current study, 52.4% of patients were diagnosed with LS based on histological analysis, with a higher prevalence being seen in the low-complexity surgical group (81.8%) compared to the high-complexity group (37.1%), although this difference did not reach statistical significance (*p* = 0.08).

Additionally, our findings revealed a statistically significant higher incidence of partial graft loss (<90%) in FTSG cases (*p* = 0.04), suggesting that, while FTSGs may offer more robust coverage, they are also associated with an increased risk of graft failure, potentially due to compromised vascularity.

We observed a 20.6% complication rate related to skin grafting, including 3.4% complete graft loss in the STSG group, 13.8% partial graft loss (primarily in the FTSG group), and 3.4% graft contracture in the FTSG group. These findings, consistent with reported complication rates of 4.7% to 33% in the literature [4,5,18,30], suggest that comorbidities such as metabolic syndrome, diabetes, and smoking may impair vascularization and increase the risk of graft failure. These results highlight the importance of preoperative measures aimed at lifestyle modification and optimal management of underlying conditions, as well as the need for improvements in techniques used for penile shaft reconstruction.

Both groups showed a significant increase in penile length from preoperative to postoperative measurements (*p* < 0.01), with a median length gain of 2 cm overall. Wahyudi et al. [36] reported a postoperative increase of 3 cm (range 0.5–7 cm) in a pediatric and young adult population following buried penis reconstruction, while Lei J. et al. [37] noted an average enhancement of 3.8 ± 0.5 cm in 64 patients using the “Six Stitch” technique for buried penis repair. However, previous studies did not specifically examine penile length changes after AABP surgery. Aligned with these studies, He et al. [38] found an increase of 2.4 ± 0.7 cm at three months post-surgery compared to preoperative measurements.

The present study also underlines the benefits of surgical intervention in the management of AABP in regard to enhancing urinary and sexual function. Unfortunately, 32.6% of patients (15/46) were unable to be contacted for questionnaire administration after surgery. Following surgery, both the IPSS and IIEF-15 scores exhibited significant improvements (*p* < 0.05) in all groups (low- and high-complexity reconstruction, FTSG and STSG groups). The overall satisfaction rate among patients was found to be 90.3%, and an improvement in QoL was registered in 93.5%. In line with these findings, Theisen et al. [12] observed improvements in both urinary and sexual function in 88% of their patients who underwent surgical repair for AABP. Similarly, Hampson et al. outlined that 74% of patients experienced a positive change in their lives after surgery [30].

The strong association between obesity and AABP observed in this study reinforces the need for effective weight management strategies as a key component of AABP prevention. As obesity rates continue to rise globally, the incidence of AABP is likely to increase, making it imperative to develop early intervention strategies for at-risk populations. Future studies should focus on preventative strategies, including weight management and early intervention in patients with mild to moderate obesity, to reduce the incidence and severity of AABP. One of the largest randomized controlled trials conducted in the USA involved 5145 overweight or obese patients with type 2 diabetes [39]. The study showed greater weight loss in the group undergoing intensive lifestyle modifications and increased physical activity compared to the group receiving diabetes support and education.

Additionally, the study highlights the need for a standardized classification system for AABP surgeries, which could facilitate better comparison of outcomes across different centers and studies. The Pariser–Santucci classification provides a useful framework, but further refinement and validation in larger, multicenter studies could improve its utility.

The main limitations of this study include its retrospective, non-randomized design, the small sample size, and its conduct at a single center. Additionally, not all patients completed the functional questionnaires. Another potential limitation is the lack of validation for the Italian version of the IIEF-15 questionnaire. Further research using a multicentric, prospective, and randomized approach is necessary to validate our findings.

## 5. Conclusions

In conclusion, our study shows that AABP surgical repair results in significant improvements in urinary function, sexual satisfaction, and overall patient satisfaction. However, the high complication rates associated with the procedure emphasize the need for careful patient selection and refinement of surgical techniques. Future research should aim to enhance surgical methods and reduce complication rates while preserving the observed functional benefits.

## Figures and Tables

**Table 1:** Baseline and perioperative characteristics of AABP patients.

Variables	Total (*n* = 46)	Low-Complexity (Santucci < III) (*n* = 11)	High-Complexity (Santucci ≥ III) (*n* = 35)	*p* Value
Nr. of patients, *n* (%)	46 (100)	11 (23.9)	35 (76.1)	0.75
Age, years (IQR)	60.5 (50.0–71.0)	60 (54.0–71.0)	61 (48.0–71. 0)	0.75
Follow-up (months), (IQR)	46 (13.0–62.0)	37 (12.0–57.0)	46 (14.0–64.0)	0.5
Previous bariatric surgery, *n* (%)	6 (13.0)	0 (0)	6 (17.1)	0.14
Risk factors and comorbidities:				
● BMI, kg/m^2^ (IQR)	30.0 (28.0–35.0)	30.0 (25.0–38.0)	31.0 (28.0–35.0)	0.48
● BMI > 25 kg/m^2^, *n* (%)	42 (95.5)	8 (72.7)	34 (97.1)	0.01
● Obesity (BMI ≥ 30 kg/m^2^), *n* (%)	27 (58.7)	6 (55.5)	21 (60.0)	0.749
● OSAS, *n* (%)	9 (19.6)	2 (18.2)	7 (20.0)	0.89
● Smoke, *n* (%)	18 (39.1)	5 (45.5)	13 (37.1)	0.72
● Hypertension, *n* (%)	24 (52.2)	6 (54.5)	18 (51.4)	0.38
● Diabetes, *n* (%)	12 (26.1)	5 (45.5)	7 (30.0)	0.09
● Previous circumcision, *n* (%)	24 (52.2)	7 (63.6)	17 (45.6)	0.38
● Previous penile cancer surgery, *n* (%)	7 (15.2)	2 (18.2)	5 (14.3)	0.75
Hospital stay (days), (IQR)	4 (3.0–5.0)	3 (1.0–5.0)	4 (3.0–5.0)	0.02
Operation time (min), (IQR)	130 (100–161)	120 (75–150)	135 (105–175)	0.28
Pariser–Santucci classification, *n* (%)				
● Category I	3 (6.5)			
● Category II	8 (17.4)			
● Category III	5 (10.9)			
● Category IV	6 (13.0)			
● Category V	24 (52.2)			
Use of skin graft, *n* (%)	29 (63.0)	8 (72.7)	21 (60.0)	0.47
Type of skin graft, *n* (%)				
● FTSG	9 (31.0)	1 (12.5)	8 (38.1)	0.18
● STSG	20 (69.0)	7 (87.5)	13 (61.9)	0.18

FTSG: Full-thickness skin graft; STSG: Split-thickness skin graft; BMI: Body Mass Index; OSAS: Obstructive Sleep Apnea Syndrome; IQR: interquartile range.

**Table 2:** Etiologies of the AABP cases and major issues reported by patients seeking medical care.

Variables	Total (*n* = 46)	Low-Complexity (Santucci < III) (*n* = 11)	High-Complexity (Santucci ≥ III) (*n* = 35)	*p* Value
Etiology, *n* (%)				
● Lichen Sclerosus	10 (21.7)	4 (36.4)	6 (17.1)	0.18
● Obesity (BMI ≥ 30 kg/m^2^)	25 (54.3)	4 (36.4)	21 (60.0)	0.17
● Previous circumcision	5 (10.9)	2 (18.1)	3 (8.6)	0.37
● Genital lymphedema/granuloma	2 (4.4)	0 (0)	2 (5.7)	0.42
● Previous penile cancer surgery	4 (8.7)	1 (9.1)	3 (8.6)	0.96
The major issue, *n* (%)				
● Poor hygiene	4 (8.7)	1 (9.1)	3 (8.6)	0.68
● Urinary issue	18 (38.1)	4 (36.4)	14 (39.9)	0.83
● Sexual issue	21 (45.7)	6 (54.5)	15 (42.9)	0.50
● Esthetic issue	3 (6.5)	0 (0)	3 (8.6)	0.32

BMI: Body Mass Index

**Table 3:** Skin grafts outcomes.

Variables	Total (*n* = 29)	FTSG (*n* = 9)	STSG (*n* = 20)	*p* Value
Operative time, min (IQR)	130 (100–161)	150 (132–190)	136 (120–175)	0.20
Donor site, *n* (%)				
● Abdomen	8 (27.6)	8 (88.9)	0 (0)	
● Thigh	20 (69)	0 (0)	20 (100)	
● Escutcheon	1 (3.4)	1 (11.1)	0 (0)	
Complete graft loss, *n* (%)	1 (3.4)	0 (0)	1 (5.0)	0.49
Partial graft loss < 90%, *n* (%)	4 (13.8)	3 (33.3)	1 (5.0)	0.04
Graft take, % (IQR)	95 (90–95)	90 (77.5–95)	95 (90–95)	0.18
Graft contracture, *n* (%)	1 (3.4)	1 (11.1)	0 (0)	0.13

FTSG: full-thickness skin graft; STSG: split-thickness skin graft; IQR: interquartile range.

**Table 4:** Surgical outcomes.

Variables	Total (*n* = 46)	Low-Complexity (Santucci < III) (*n* = 11)	High-Complexity (Santucci ≥ III) (*n* = 35)	*p* Value
Postoperative complications, nr. patients (%)	15 (32.6)	2 (18.2)	13 (37.1)	0.24
Clavien classification, *n* (%)				
● I	7 (46.7)	1 (50.0)	6 (46.2)	
● II	6 (40.0)	1 (50.0)	5 (38.5)	
● IIIb	2 (13.3)	0 (0)	2 (15.4)	
Recurrence rate, *n* (%) *	10 (21.7)	3 (27.3)	7 (20.0)	0.66
RFS rate at 12 months FU, *n* (%)	41 (89.1)	10 (90.9)	31 (88.6)	0.85
Presence of LS at histology, *n* (%)	22 (52.4)	9 (81.8)	13 (37.1)	0.08
Preop penile length, cm (IQR)	7.0 (6.0–9.6)	7 (4–10.5)	7 (6.0–9.3)	0.79
Post penile length, cm (IQR)	11.3 (8.5–13.0)	12 (9.0–13.0)	10 (8.3–12.5)	0.49
Difference in penile length, cm (IQR)	2.0 (1.5–3.8)	2.5 (2.0–6.0)	2 (1.5–3.0)	0.14

* The need for any surgical intervention due to re-burying of the penis at follow-up. RFS rate: recurrence-free survival rate; FU: follow-up; IQR: interquartile range.

**Table 5:** Functional outcomes (31 patients): urinary and sexual functions.

	IPSS Pre	IPSS Post	*p* Value
Total score, *n* (IQR)	10.0 (3.5–14.0)	3.0 (1.0–7.0)	<0.001
Low-complexity (Santucci < III), *n* (IQR)	8.0 (2.5–13.0)	3.0 (0.0–7.0)	0.04
High-complexity (Santucci ≥ III), *n* (IQR)	10.0 (3.0–15.5)	3.0 (1.0–7.5)	<0.001
	IIEF-15 Pre	IIEF-15 Post	*p* Value
Total score, *n* (IQR)	31.0 (15.0–63.0)	57.0 (29.0–70.0)	<0.001
Low-complexity (Santucci < III), *n* (IQR)	25.0 (15.0–54.5)	65.0 (26.0–70.0)	0.02
High-complexity (Santucci ≥ III), *n* (IQR)	34.0 (15.5–64.0)	53.5 (29.0–70.0)	<0.001
FTSG use, *n* (IQR)	38.0 (21.0–72.0)	59.0 (35.0–73.0)	0.04
STSG use, *n* (IQR)	17.0 (8.0–33.0)	29.0 (13.0–68.0)	<0.001

FTSG: full-thickness skin graft; STSG: split-thickness skin graft; IPSS: international prostate symptom score; IIEF-15: International Index of Erectile Function; IQR: interquartile range.

**Table 6:** Patient-reported outcomes of the group following surgery.

Variables	Total (*n* = 31)	Low-Complexity (Santucci < III) (*n* = 7)	High-Complexity (Santucci ≥ III) (*n* = 24)	*p* Value
Overall satisfaction for the operation, *n* (%)	28 (90.3)	7 (100)	21 (87.5)	0.32
Overall improvement of urinary function, *n* (%)	31 (100)	7 (100)	24 (100)	1
Overall improvement of genital hygienic care, *n* (%)	30 (96.8)	7 (100)	23 (95.8)	0.58
Would you suggest a friend undergo the same operation? *n* (%)	29 (93.5)	6 (85.7)	23 (95.8)	0.34
Improvement of sexual life after surgery, *n* (%)	20 (66.7)	6 (85.7)	14 (60.9)	0.22
Positive impact of surgery on quality of life, *n* (%)	29 (93.5)	7 (100)	22 (92.7)	0.43

## Data Availability

The original contributions presented in the study are included in the article, further inquiries can be directed to the corresponding author.

## Appendix A

**Table A1:** Ad “hoc” 6-items questionnaire.

	YES	NO
1. Overall, are you satisfied with the intervention?		
2. Did the surgery have a positive impact on the quality of your urinary function?		
3. Did the surgery improve your genital hygiene?		
4. Would you recommend the surgery to a friend/family member?		
5. Did the surgery improve your quality of life?		
6. Did the surgery have a positive impact on the quality of your sexual life?		
